# School day segmented physical activity patterns of high and low active children

**DOI:** 10.1186/1471-2458-12-406

**Published:** 2012-06-06

**Authors:** Stuart J Fairclough, Aaron Beighle, Heather Erwin, Nicola D Ridgers

**Affiliations:** 1 Research Institute for Sport and Exercise Sciences, Liverpool John Moores University, Tom Reilly Building, Byrom Street, Liverpool, UK; 2 Department of Kinesiology and Health Promotion, University of Kentucky, Kentucky, USA; 3 Centre for Physical Activity and Nutrition Research, Deakin University, Deakin, Australia

**Keywords:** Youth, Moderate physical activity, Vigorous physical activity, Accelerometer, Multi-level analyses, Segments

## Abstract

**Background:**

Variability exists in children’s activity patterns due to the association with environmental, social, demographic, and inter-individual factors. This study described accelerometer assessed physical activity patterns of high and low active children during segmented school week days whilst controlling for potential correlates.

**Methods:**

Two hundred and twenty-three children (mean age: 10.7 ± 0.3 yrs, 55.6% girls, 18.9% overweight/obese) from 8 north-west England primary schools wore ActiGraph GT1M accelerometers for 7 consecutive days during autumn of 2009. ActiGraph counts were converted to minutes of moderate (MPA), vigorous (VPA) and moderate-to-vigorous (MVPA) physical activity. Children were classified as high active (HIGH) or low active (LOW) depending on the percentage of week days they accumulated at least 60 minutes of MVPA. Minutes spent in MPA and VPA were calculated for school time and non-school time and for five discrete school day segments (before-school, class time, recess, lunchtime, and after-school). Data were analysed using multi-level modelling.

**Results:**

The HIGH group spent significantly longer in MPA and/or VPA before-school, during class time, lunchtime, and after-school (*P* < .05), independent of child and school level factors. The greatest differences occurred after-school (MPA = 5.5 minutes, VPA = 3.8 minutes, *P* < 0.001). MPA and VPA were also associated with gender, BMI z-score, number of enrolled children, playground area per student, and temperature, depending on the segment analysed.

The additive effect of the segment differences was that the HIGH group accumulated 12.5 minutes per day more MVPA than the LOW group.

**Conclusions:**

HIGH active children achieved significantly more MPA and VPA than LOW active during four of the five segments of the school day when analyses were adjusted for potential correlates. Physical activity promotion strategies targeting low active children during discretionary physical activity segments of the day, and particularly via structured afterschool physical activity programs may be beneficial.

## Background

Evidence regarding the benefits of regular physical activity for youth continues to mount but in many countries physical activity engagement remains below levels deemed necessary to receive many of these benefits [1]. In the United States, for example, 42% of boys and girls aged 6–12 years and only 8% of boys and girls aged 12–19 years are meeting national recommendations of 60 minutes or more of at least moderate intensity physical activity (MPA) every day [2]. Similarly, in England the proportion of 4 to 10 yr old boys and girls achieving this guideline when physical activity is assessed objectively is 51% and 34%, respectively [3], and 7% (boys) and 0% (girls) among 11 to 15 yr olds [3]. Consequently, there is a need for comprehensive multi-level interventions to address the current status of youth physical activity.

One approach with much promise is school-based, multi-faceted strategies that focus on both in school and out of school physical activity [4]. Schools offer youth a structured environment with formal (e.g., physical education classes, after-school clubs) and informal (e.g., recess, active travel) opportunities for physical activity. Although schools are attractive settings for physical activity participation, data focused on physical activity during specific segments of school week days are limited [5,6] with few studies using accelerometry [7-9], and even fewer controlling analyses for known confounders and correlates [9]. Previous investigations have shown the influences of biological [10], environmental [11], and demographic factors [12] on children’s physical activity. To our knowledge only two other studies of activity patterns during the segmented school week have controlled for the effects of such physical activity correlates [9,13]. Both of these were observational studies which sampled UK 9–10 year olds. Steele and colleagues assessed sedentary time, and vigorous physical activity during school time, out of school, on week days and at weekends, and adjusted their analyses for anthropometric and demographic child-level correlates [9].

Ridgers et al. investigated correlates most strongly related to moderate-to-vigorous physical activity during school, after-school, on week days and at weekends [11]. The prediction models used in analyses included anthropometric, demographic, fitness, behavioural, and local environment child-level correlates [11]. The current study builds on this research by incorporating a range of child-level correlates with school-level variables which were hypothesised to influence physical activity during discrete segments of the school day.

Theoretically schools provide all students with the same environmental stimuli to be physically active, but in reality physical activity engagement is highly variable between individuals across whole school days and during specific segments [6,7]. It is unclear though to what extent youth physical activity differs during the same segments of the day, and which segments offer most potential for physical activity engagement. A recent systematic review suggested that the more physical activity youth engage in, the greater the health benefit [14], thus, investigating low and high active children’s physical activity within segmented school week days is warranted from a health perspective. Furthermore, identifying child and school-level factors that influence accumulation of physical activity in children may help in the design of more effective interventions. Therefore, the aims of this study were (1) to describe accelerometer assessed physical activity patterns during segmented school week days, (2) to investigate the magnitude of differences in physical activity between high and low active children during segments of school week days, and (3) to investigate associations between child and school-level correlates with the physical activity of high and low active children during segments of school week days.

## Methods

### Study design and participants

Eight geographically representative schools situated in Wigan, north-west England expressed an interest in the study and were recruited during the autumn school term of 2009. Wigan is a mixed urban and rural municipal borough with a population of over 300,000 [15]. Ethical approval was obtained from the Liverpool John Moores University Research Ethics Committee, and in the eight schools all 295 children in Grade 6 (aged 10―11 years) received verbal and written project information and were invited to participate in the study. Signed informed parental consent and child assent were received from 269 children (138 girls) resulting in a 91.2% participation rate. Parents also completed a brief questionnaire which collected demographic information (i.e., age, sex, ethnicity, home post code). The ethnic origin of the consenting children was 98.9% white British, 0.4% white European, and 0.7% Asian, which roughly reflects the ethnic demographic of the borough’s school-age population. Data were collected in one school per week between October and December 2009.

### Instruments

Physical activity was objectively assessed using ActiGraph uniaxial accelerometers (GT1M, ActiGraph LLC, Pensacola, FL). The ActiGraph is a small (3.8 x 3.7 x 1.8 cm), lightweight (27 g) monitor designed to detect vertical accelerations ranging in magnitude from 0.05 to 2.00g, with a frequency response of 0.25–2.50 Hz. Five second epochs were used to accurately capture children’s intermittent activity patterns [16]. The ActiGraph has acceptable validity and reliability for use in paediatric studies [17], and it is the most commonly used accelerometer in field-based research.

### Procedures

Physical activity. As the children had no prior experience of wearing accelerometers they received a brief familiarization session where they were shown how to attach and remove the adjustable elastic ActiGraph belt and the correct positioning of the instrument over the right hip. Instructions were given regarding ActiGraph removal (only during water-based activities, contact sports where risk of injury or monitor damage was high, and sleeping), and replacement (on waking in the morning, after any other occurrences of removal). The children were encouraged to ignore the instrument whilst wearing it and go about their normal activities during the monitoring period. The ActiGraphs were set to record data for 7 consecutive days (Friday through Friday), though for the purposes of this study only week day data were used.

Child-level correlates. Home post codes were used to generate indices of multiple deprivation (IMD) scores which indicate neighbourhood socio-economic status (SES). IMD scores are a composite of seven domains of deprivation (income, employment, education, health, crime, access to services, and living environment) [18] with higher scores representing higher degrees of deprivation. Stature and sitting height were measured to the nearest 0.1 cm using a portable stadiometer (Leicester Height Measure, Seca, Birmingham, UK). Leg length was calculated by subtracting sitting height from stature. Body mass was measured to the nearest 0.1 kg using calibrated scales (Seca, Birmingham, UK). All measurements were taken by trained research staff with the children in light clothing and barefooted. Body mass index (BMI) was calculated (body mass (kg)/stature^2^ (m^2^)) and BMI z-scores were assigned to each participant [19]. International Obesity Task Force age and sex-specific BMI cut-points [20] were used to classify children as either normal-weight, overweight, or obese. Somatic maturity status was estimated by maturity offset values (i.e., years from attainment of peak height velocity [APHV]), which were calculated using sex-specific regression equations that included stature, sitting height, leg length, chronological age, body mass (girls only), and their interactions [21].

School-level correlates. The number of children enrolled in each school was recorded. Aerial views of the schools’ playground areas were located using the Google™ Earth Pro (GEP) application (version 4.2.0205.5730). Playground areas were calculated using the GEP polygon tool and summed for each school, to provide an estimate of playground spatial area [11]. During the data collection period daily temperature and number of days rainfall per week were recorded [22]. This combination of variables was selected based on previous research that showed significant associations with elementary school children’s physical activity [10-13].

### Data reduction

At the end of the monitoring period ActiGraphs were downloaded using Actilife v5 software (ActiGraph LLC, Pensacola, FL) and initially checked for compliance to the monitoring protocol using customized software (MeterPlus v4.2, Santech Inc., San Diego, CA; http://www.meterplussoftware.com). Sustained 20 minute periods of zero counts indicated that the ActiGraph had been removed, and total ‘missing’ counts for those periods represented the duration that monitors were not worn [23]. Children were included in the data analysis if they wore the monitors for at least 600 minutes each day for a minimum of three week days [24]. ActiGraph count cut-points of 2000 and 4000 counts · min^−1^ represented time spent in MPA and vigorous physical activity (VPA), respectively [25]. The lower limit for MPA corresponds to a walking pace of around 3–4 km/h in 10–14 year olds [17], and was recently used to study associations between PA intensity and adiposity in English 10 year olds [26]. To accommodate the 5 second epoch length, the count cut-point thresholds were divided by 12. MPA and VPA were calculated by summing minutes spent in each activity threshold during five discrete segments of the day: before-school (8.30–9am), class time, recess, lunchtime, and after-school (e.g., 3.30–6.30pm). The segment before school could include time at home before the morning commute to school, the commute itself, and time at school before the school day formally commenced. Recess and lunchtime periods are mandatory in English schools. They are generally peer-controlled with minimal adult supervision, (i.e., playground supervisors are present only to manage general playground behaviour). Recess takes places on outdoor playgrounds, which tend to have some form of floor markings to encourage active play. During lunchtimes children are allowed to go out onto the playground after eating indoors. On rainy days recess and lunchtime activities generally take place in classrooms. All schools had morning recess and three had afternoon recess, and physical activity during recess periods was summed to calculate total recess physical activity. Physical Education (PE) classes were scheduled twice per week in each school, but the actual frequency and duration of classes and mode of activities were very inconsistent between schools due to irregular events such as class assemblies, extended classroom lessons, and wet weather. Due to the low number of PE classes PE time was not included as a discrete segment but instead was integrated within class time activity. After school some school children walk, cycle, or are transported home or to another destination immediately that schools ends, while others remain in school for a structured physical activity or sport club.

### Data analysis

Mean minutes of MPA and VPA were calculated for the whole day, school time, out of school time, and for each segment. Total time spent in moderate to vigorous physical activity (MVPA) on each of the five week days was calculated for each child, and using the 60 minutes MVPA per day minimum recommendation for health, children were dichotomized into two groups. Low active children (LOW) were categorised as those who achieved this recommendation on < 50% of their valid days, while high active children (HIGH) achieved it for ≥ 50% of their valid days [27]. Descriptive analyses were initially conducted to explore group differences in anthropometric and physical activity data using factorial ANOVAs and Chi Square tests. These analyses were carried out using SPSS version 17 (SPSS Inc., Chicago, IL) with alpha set at *P* < .05 and mean (SD) data reported.

The main analysis consisted of multilevel analyses to account for the nested nature of the child data within the eight schools. A two-level data structure was used where children were defined as the first level unit of analysis and schools as the second level unit [28]. Schools were included as a second level unit to control for the effect that this particular context could have on the children’s physical activity [28]. Data were analyzed using MLwiN 2.20 software (Centre for Multi-Level Modelling, University of Bristol, UK). Association models were used to assess the effects of the physical activity groups (HIGH, LOW) on the outcome variables of MPA and VPA time during school time, out of school time, and the five discrete segments that occurred within these periods. Analyses were conducted with adjustment made for child level correlates (sex, chronological age, BMI z-score, somatic maturity, IMD score), and school level correlates (segment duration, number of enrolled students per school, school playground area per student, average daily temperature, average weekly rainfall), which were identified a priori based on previous research [10-13]. Regression coefficients in the models were assessed for significance using the Wald statistic [28] and the alpha level was set at *P* < .05.

## Results

### Exploratory analyses

Forty-six children (17.1%) did not meet the minimum ActiGraph wear time criteria due to monitor malfunction (n = 12) and non-compliance (n = 34). Overall, 223 children were included in the analyses (124 girls) giving an 82.9% compliance rate. Significant differences between included and excluded children were observed for somatic maturity (included > excluded, *P* < .01). Ninety eight children (43.9%) were categorised as high active (HIGH), and 125 (56.1%) were low active (LOW). Mean duration of daily monitoring was 723.8 ± 80.2 minutes and 692.1 ± 84.9 minutes for HIGH and LOW groups, respectively. Descriptive characteristics of the groups are presented in Table 1. In both groups girls were somatically more mature than boys (*P* < .001). The HIGH group recorded significantly more unadjusted MPA and VPA than the LOW group (*P* < .05) for each segment with the exception of beforeschool VPA and recess.

**Table 1:** Descriptive characteristics and unadjusted physical activity data of participants (M ± SD except weight status)

	**HIGH active**			**LOW active**		
Demographic variables	All (n = 98)	Boys (n = 58)	Girls (n = 40)	All (n = 125)	Boys (n = 41)	Girls (n = 84)
Age (yr)	10.6 (0.3)	10.6 (0.4)	10.7 (0.3)	10.7 (0.3)	10.7 (0.3)	10.7 (0.3)
IMD score	17.6 (10.3)	17.5 (9.9)	17.7 (10.9)	18.8 (11.7)	18.3 (13.3)	18.7 (10.6)
Anthropometric variables						
Stature (cm)	142.8 (7.0)	142.4 (7.0)	143.4 (7.3)	143.8 (7.7)	141.5 (7.3)	143.4 (7.3)
Body mass (kg)	35.7 (7.7)	35.5 (7.6)	35.8 (8.0)	38.9 (9.3)*	36.5 (9.1)	40.2 (9.2)
BMI z-score	−0.01 (1.2)	0.1 (1.1)	−0.2 (1.3)	0.4 (1.3)*	0.3 (1.5)	0.4 (1.2)
Maturity offset (yr)	−2.4 (1.0)	−3.1 (0.5)	−1.3 (0.6)**	−1.8 (1.1)	−3.1 (0.5)	−1.2 (0.6)**
Weight status variables						
% Normal weight	87.8%	87.9%	87.5%	75.8%	80.5%	73.5%
% Overweight	10.2%	8.6%	12.5%	18.5%	17.1%	19.3%
% Obese	2.0%	3.4%	0.0%	5.6%	2.4%	7.2%
Physical activity variables						
PA count·min^-1^	594.3 (131.5)	610.4 (150.6)	569.6 (91.6)	414.0 (75.8)	422.5 (68.4)	409.5 (79.5)
Daily MPA (min)	43.7 (12.2)	45.8 (12.0)	40.5 (11.8)	35.7 (8.6)	36.3 (8.5)	35.4 (8.6)
Daily VPA (min)	23.2 (9.7)‡	24.2 (10.0)	21.8 (9.1)	16.9 (6.4)	18.2 (6.4)	16.2 (6.4)
School time MPA (min)	17.6 (4.9)‡	18.9 (4.7)	15.6 (4.4)	14.8 (4.4)	15.0 (3.8)	14.7 (4.6)
School time VPA (min)	10.8 (4.0)‡	11.2 (4.2)	10.2 (3.6)	8.5 (3.9)	9.0 (3.1)	8.2 (4.3)
Out of school MPA (min)	26.1 (9.6)‡	27.1 (9.7)	24.6 (9.4)	21.1 (6.4)	21.7 (7.0)	20.8 (6.1)
Out of school VPA (min)	12.3 (7.0)‡	13.0 (7.2)	11.3 (6.8)	8.6 (3.8)	9.6 (4.4)	8.0 (3.3)
Before school MPA (min)	3.8 (2.8)††	3.6 (2.2)	4.1 (3.4)	3.0 (1.8)	2.9 (1.7)	3.1 (1.9)
Before school VPA (min)	1.2 (1.3)	1.3 (1.2)	0.9 (1.5)	0.8 (0.9)	0.9 (1.1)	0.7 (0.8)
Class time MPA (min)	10.3 (3.3)†	11.2 (3.4)	8.9 (2.6)	9.0 (2.9)	9.1 (2.9)	8.9 (2.9)
Class time VPA (min)	6.3 (2.4)†	6.6 (2.6)	5.8 (2.1)	5.3 (2.5)	5.8 (2.3)	5.1 (2.6)
Recess MPA (min)	1.3 (0.8)	1.2 (0.8)	1.4 (0.9)	1.2 (0.9)	1.3 (0.7)	1.2 (0.9)
Recess VPA (min)	0.9 (0.7)	0.8 (0.7)	1.0 (0.8)	0.8 (0.8)	0.9 (0.8)	0.8 (0.8)
Lunchtime MPA (min)	6.0 (2.3)	6.5 (2.3)	5.3 (2.0)	4.6 (2.0)	4.5 (1.9)	4.6 (2.1)
Lunchtime VPA (min)	_3.6 (2.3)‡_	3.8 (2.4)	3.4 (2.0)	2.3 (1.8)	2.3 (1.7)	2.3 (1.9)
After school MPA (min)	15.2 (5.4)‡	15.8 (5.5)	14.2 (5.1)	10.4 (3.5)	9.8 (3.5)	10.7 (3.5)
After school VPA (min)	7.9 (4.6)‡	8.2 (4.8)	7.4 (4.3)	3.7 (1.9)	3.4 (1.7)	3.8 (2.0)

BMI: body mass index; IMD: Indices of Multiple Deprivation; MPA: moderate intensity physical activity; VPA: vigorous intensity physical activity;

* LOW > HIGH, *P* < .05.

** HIGH girls > HIGH boys; LOW girls > LOW boys, *P* < .001 † HIGH > LOW, *P* < .05.

†† HIGH > LOW, *P* < .01.

‡ HIGH > LOW, *P* < .001.

### Main analyses

School level variables entered into the multi level models are presented in Table 2. The multilevel analyses are reported in Tables 3, 4, 5, 6. The overall models accounted for 6.5%–33% and 14.4%–43.4% of the variance in MPA and VPA, respectively, depending on the segment analysed. The percentage of model variance explained by group, child, and school-level correlates varied depending on school day segment (Tables 3, 4, 5, 6). In the after-school segment, physical activity group explained 22.9% and 28.1% of variance in MPA and VPA, respectively, but less than 3% at recess and before-school. The anthropometric and demographic child level correlates explained most variance after-school (MPA: 11.1%, VPA: 21.9%) and least at lunchtime (MPA: 0.7%, VPA: 1.5%). The greatest proportion of variance explained by school level correlates was during recess and lunchtime (9%–20.1%), with the smallest proportion being after-school (1.1%–3.4%).

**Table 2:** Descriptive school level variables

**Variable**	**Mean (SD)**	**Range**
No. enrolled students	357.3 (141.5)	149-517
Playground area (m^2^)	1951.7 (1042.3)	581-3669
Playground area student (m^2^ student)	5.4 (1.5)	7.6-2.8
Average temperature (^°^C)	8.6 (2.4)	3.7-12.0
No. days rainfall	2.3 (0.7)	1.0-3.0
Segment duration (min)		
Before-school	30.0 (0.0)	N/A
Class time	300.4 (12.7)	285.0-330.0
Recess	18.8 (6.2)	15.0-30.0
Lunchtime	60.7 (4.8)	55.0-75.0
After-school	126.7 (6.7)	120.0-135.0

**Table 3:** School time and out of school time multilevel analysis of MPA and VPA

	**School time**		**Out of school time**	
	MPA β (SE)	VPA β (SE)	MPA β (SE)	VPA β (SE)
Constant	8.3 (46.1)	−3.4 (26.7)	2.8 (65.7)	−13.2 (39.2)
Physical activity group^a^	1.6 (0.6)††	1.4 (0.5)††	5.1 (1.1)‡	3.0 (0.7)‡
% variance explained	10.3	9.4	10.4	11.2
Child level variables				
Sex^b^	−0.1 (1.3)	1.4 (1.2)	0.6 (2.5)	2.7 (1.7)
Age	0.9 (1.0)	−0.04 (0.9)	−0.8 (1.9)	0.6 (1.3)
BMI z-score	0.4 (0.3)	−0.3 (0.2)	1.1 (0.5)†	−0.3 (0.3)
Maturity offset (years from APHV)	−1.0 (0.7)	0.1 (0.6)	−0.5 (1.3)	0.6 (0.9)
IMD	0.02 (0.03)	0.01 (0.03)	0.03 (0.1)	0.05 (0.04)
% variance explained	3.4	3.4	10.8	18.8
School level variables				
Duration (min)	−0.01 (0.1)	0.03 (0.06)	0.03 (0.1)	0.03 (0.1)
No. enrolled students	−0.01 (0.01)	−0.01 (0.004)	0.01 (0.01)	0.002 (0.004)
Playground area (m^2^·student^-1^)	1.0 (0.5)	0.8 (0.3)††	0.4 (0.5)	0.3 (0.3)
Average temperature (^°^C)	0.01 (0.1)	−0.1 (0.1)	0.2 (0.3)	−0.2 (0.2)
No. days rainfall	−0.2 (0.5)	−0.3 (0.5)	−0.1 (1.1)	−0.6 (0.7)
% variance explained	18.5	15.3	15.3	3.7
Overall model				
% Total variance explained	28.5	23.0	25.0	27.4
School level variance	4.7 (2.6)	1.1 (0.8)	2.5 (2.3)	0.5 (0.7)
Child level variance	14.3 (1.4)	12.5 (1.2)	53.0 (5.3)	24.7 (2.5)
Total variance	19.0	13.6	55.5	25.2
Deviance	1222.5	1183.7	1415.4	1254.4
ICC	0.25	0.08	0.04	0.02

^a^For Physical activity group the reference category is LOW. ^b^For Sex the reference category is girls. β (SE)

values rounded to 1 decimal place for presentation purposes.

†*P* < .05.

††*P* < .01.

‡*P* < .001.

**Table 4:** Before-school and class time multilevel analysis of MPA and VPA

	**Before-school**		**Class time**	
	MPA β (SE)	VPA β (SE)	MPA β (SE)	VPA β (SE)
Constant	3.8 (6.4)	−1.6 (2.9)	−5.0 (18.5)	2.4 (13.1)
Physical activity group^a^	0.9 (0.3)††	0.2 (0.1)	0.7 (0.3)†	0.4 (0.3)
% variance explained	2.8	3.3	6.0	4.7
Child level variables				
Sex^b^	−1.01 (0.8)	0.3 (0.3)	−0.4 (0.8)	0.6 (0.7)
Age	−0.04 (0.6)	0.2 (0.3)	0.8 (0.6)	0.3 (0.5)
BMI z-score	0.2 (0.1)	−0.1 (0.1)	0.3 (0.2)	−0.1 (0.1)
Maturity offset (years from APHV)	−0.4 (0.4)	0.02 (0.2)	−0.8 (0.4)	−0.1 (0.3)
IMD	0.01 (0.02)	−0.001 (0.01)	0.01 (0.02)	0.01 (0.01)
% variance explained	1.0	6.0	3.4	4.5
School level variables				
Duration (min)	0.0001 (0.0001)	0.0001 (0.0001)	0.02 (0.1)	−0.001 (0.04)
No. enrolled students	0.002 (0.001)†	0.001 (0.001)	−0.01 (0.01)	−0.003 (0.004)
Playground area (m^2^·student^-1^)	0.1 (0.1)	0.15 (0.06)†	05 (0.5)	0.4 (0.3)
Average temperature (^°^C)	−0.1 (0.9)	−0.1 (0.04)	−0.1 (0.1)	−0.1 (0.1)
No. days rainfall	−0.5 (0.3)	−0.2 (0.1)	−0.1 (0.3)	−0.3 (0.3)
% variance explained	3.0	10.7	13.8	9.0
Overall model				
% Total variance explained	6.5	17.4	20.3	14.4
School level variance	0.0	0.03 (0.03)	3.9 (2.1)	1.8 (1.0)
Child level variance	4.9 (0.5)	1.0 (0.1)	5.3 (0.5)	3.7 (0.4)
Total variance	4.9	1.0	9.2	5.5
Deviance	961.7	620.3	1010.9	928.9
ICC	0.0	0.03	0.42	0.32

^a^For Physical activity group the reference category is LOW. ^b^For Sex the reference category is girls. β (SE)

values rounded to 1 decimal place for presentation purposes.

†*P* < .05.

††*P* < .01.

**Table 5:** Recess and lunchtime multilevel analysis of MPA and VPA

	**Recess**		**Lunchtime**	
	MPA β (SE)	VPA β (SE)	MPA β (SE)	VPA β (SE)
Constant	−1.4 (4.7)	−1.6 (4.1)	−1.6 (7.0)	−2.2 (6.7)
Physical activity group^a^	0.3 (0.2)	0.2 (0.2)	0.8 (0.3)††	0.8 (0.2)††
% variance explained	1.1	1.6	9.7	10.8
Child level variables				
Sex^b^	1.4 (0.5)††	0.9 (0.5)	0.4 (0.6)	0.9 (0.6)
Age	0.1 (0.4)	0.2 (0.3)	−0.02 (0.5)	−0.04 (0.4)
BMI z-score	−0.1 (0.1)	−0.2 (0.1)†	0.1 (0.1)	−0.1 (0.1)
Maturity offset (years from APHV)	0.5 (0.3)	0.3 (0.2)	−0.1 (0.3)	0.2 (0.3)
IMD	0.0001 (0.01)	−0.01 (0.01)	0.01 (0.01)	−0.0001 (0.01)
% variance explained	8.9	4.0	0.7	1.5
School level variables				
Duration (min)	0.1 (0.1)	0.05 (0.04)	0.1 (0.1)	0.1 (0.1)
No. enrolled students	0.004 (0.002)†	0.002 (0.002)	−0.002 (0.003)	−0.002 (0.003)
Playground area (m^2^·student^-1^)	0.3 (0.2)	0.3 (0.2)	0.5 (0.2)†	0.3 (0.2)
Average temperature (^°^C)	_−0.2 (0.1)††_	_−0.2 (0.1)‡_	0.05 (0.1)	−0.03 (0.1)
No. days rainfall	−0.1 (0.2)	−0.2 (0.2)	−0.1 (0.3)	−0.3 (0.3)
% variance explained	20.1	13.0	18.9	17.1
Overall model				
% Total variance explained	26.5	18.3	28.5	26.5
School level variance	0.5 (0.3)	0.3 (0.2)	0.7 (0.4)	0.7 (0.4)
Child level variance	2.2 (0.2)	1.7 (0.2)	3.2 90.3)	2.8 (0.3)
Total variance	2.7	2.0	3.9	3.5
Deviance	808.3	756.3	889.5	862.4
ICC	0.19	0.16	0.18	0.21

^a^For Physical activity group the reference category is LOW. ^b^For Sex the reference category is girls. β (SE)

values rounded to 1 decimal place for presentation purposes.

†*P* < .05.

††*P* < .01‡*P* < .001.

**Table 6:** After-school multilevel analysis of MPA and VPA

	**MPA β (SE)**	**VPA β (SE)**
Constant	13.2 (15.1)	4,1 (10.3)
Physical activity group^a^	4.5 (0.6)‡	3.8 (0.4)‡
% variance explained	22.9	28.1
Child level variables		
Sex^b^	0.3 (1.4)	0.1 (1.0)
Age	−0.9 (1.0)	−0.3 (0.8)
BMI z-score	−0.04 (0.3)	−0.4 (0.2)†
Maturity offset (years from APHV)	0.1 (0.7)	0.02 (0.5)
IMD	0.04 (0.03)	0.04 (0.02)
% variance explained	11.1	21.9
School level variables		
Duration (min)	0.02 (0.1)	0.02 (0.04)
No. enrolled students	0.01 (0.004)	0.001 (0.002)
Playground area (m^2^ student^-1^)	−0.07 (0.3)	−0.1 (0.2)
Average temperature (^°^C)	0.1 (0.2)	0.05 (0.1)
No. days rainfall	0.5 (0.6)	0.2 (0.4)
% variance explained	3.4	1.1
Overall model		
% Total variance explained	33.0	43.4
School level variance	1.0 (0.8)	0.1 (0.2)
Child level variance	15.7 (1.5)	8.6 (0.8)
Total variance	16.7	8.7
Deviance	1227.0	1091.1
ICC	0.06	0.01

^a^For Physical activity group the reference category is LOW. ^b^For Sex the reference category is girls. β (SE)

values rounded to 1 decimal place for presentation purposes.

†*P* < .05.

‡*P* < .001.

The HIGH group engaged in 1.6 ± 0.6 (*P* < .01) and 5.1 ± 1.1 (*P* < .001) more minutes of MPA than the LOW group during school time and out of school time, respectively. For VPA, the HIGH group accrued 1.4 ± 0.5 (*P* < .01) and 3.0 ± 0.7 (*P* < .001) more minutes than the LOW group during school time and out of school time, respectively (Table 3). In reference to specific segments of the day, the HIGH group engaged in more MPA (0.9 ± 0.3 minutes; *P* < .01) before-school and during class time (0.7 ± 0.3 minutes; *P* < .05) than the LOW group (Table 4). Furthermore, before-school MPA and VPA were positively associated with number of enrolled students (*P* < .05) and playground area per student (*P* < .05), respectively.

There were no significant between-activity group differences in MPA or VPA during recess but at lunchtime the HIGH group accumulated more activity at both intensities (*P* < .01; Table 5). Boys spent more time (1.4 ± 0.5 minutes) than girls in recess MPA (*P* < .01), which also was significantly associated with student enrolment (*P* < .05). Temperature was inversely associated with recess MPA (*P* < .01) and VPA (*P* < .001). Lunchtime MPA was positively associated with playground area per student (*P* < .05). The greatest group differences in MPA and VPA occurred in the after-school segment (Table 6). The HIGH group spent 4.5 ± 0.6 minutes (*P* < .001) and 3.8 ± 0.4 minutes (*P* < .001) more in MPA and VPA, respectively than the low group. During this segment VPA was inversely associated with BMI z-scores (*P* < .05).

## Discussion

### In school and out of school

This study is the first to investigate high and low active children’s segmented school day physical activity patterns using accelerometry whilst controlling for effects of influential correlates. Children were most active out of school and the greatest differences in MPA and VPA between the HIGH and LOW active groups occurred out of school. This is likely due to the greater discretionary time available for physical activity and other recreational pursuits during non-school hours, and is consistent with other studies using objective physical activity measures [5-7,9]. In agreement with our findings Cox and colleagues observed significantly more pedometer assessed physical activity out of school among the most active children (55.1% vs. 44.9% of total daily steps), but for the least active children most steps were accrued in school (53.3% vs. 46.7%) [29]. These authors suggested that there is a ceiling to the amount of physical activity possible during school and that outside of school more time, choice, and opportunities for physical activity are available, which are better exploited by the most active children [29]. The school day structure and discretionary nature of out of school time can be both barriers and enabling factors for physical activity. The HIGH (57.5%) and LOW (56.5%) active groups though accumulated most of their physical activity out of school, suggesting that the constraining structure of the school day impacted similarly on both groups’ MPA and VPA.

### Before-school

During the 30 minute segment before school the HIGH group achieved more MPA than the LOW group, which concurs with previous findings [30]. Although other studies [7,13,31,32] have also assessed MVPA in the before-school period differences in segment durations and accelerometer count cut-points negate meaningful comparisons. Associations between higher physical activity levels before school and active commuting have previously been reported [33], though the HIGH group may also have engaged in more playground activity once they arrived at school. It has been observed that children are more active during the morning walk to school compared to the period in the playground before school begins [31], but once in the playground the most active children may be more predisposed to engage in MPA and VPA than less active peers.

### Recess

Temperature was inversely associated with recess MPA and VPA, which is consistent with previously reported findings from studies conducted in north-west England [11]. In this region during autumn and winter low temperatures tend to be accompanied by dryer weather, while milder temperatures are associated with wetter weather. During cold weather children are outside during recess, and it is plausible that they may indirectly engage in playground activity to produce body heat. Conversely, a large study of pedometer determined physical activity in New Zealand found that reduction in mean temperature had a small, but negative impact on activity levels [34]. These authors concluded that the influence of weather on youth physical activity is not consistent for all populations, but strategies for physical activity engagement are recommended on cold and/or rainy days. As north-west England is often cold and wet in winter, such strategies would be particularly relevant to youth in this location. During the study, on average it rained for 2.3 days of the 5 day school week. A recent UK investigation showed that whole day as well as lunchtime physical activity levels were lower on rainy days compared to dry ones [35]. The authors suggested that school policies in relation to wet recess periods may influence activity levels, because on the wettest days children allowed outside at lunchtime spent significantly less time in MVPA than those who stayed indoors but were allowed to be active [35]. On rainy days the children in our study were not allowed outside at recess, with the alternative being quiet classroom reading and socialising. Recess physical activity was calculated based on the scheduled recess timings in each school, regardless of whether the children were indoors or outside, thus it is possible that reduced time outdoors due to rain contributed to the relatively low levels of recess physical activity observed. Where children are confined indoors during wet recess periods, provision should be made for these periods to enable at least light and preferably moderate intensity activities, rather than an emphasis on sedentary ones.

Boys spent significantly more time in recess MPA than girls, which is consistent with other studies which have suggested that differentiated gender roles exist at recess, with boys viewing it as an opportunity to engage in competitive games, and girls spending more time socialising with friends [36]. The magnitude of the sex differences in recess MPA was smaller than has previously been reported [36] most likely because the analyses controlled for maturation, which is a highly influential variable when physical activity is compared between boys and girls of similar chronological age [10].

### Lunchtime

During the lunchtime segment there were modest, yet significant group differences in MPA and VPA. Mean lunchtime duration was 61.6 minutes but because of the different organizational routines used in the schools the amount of time the children had for outdoor play during the lunch period varied. The significant group differences in MPA and VPA observed during lunchtime but not during recess suggests that the longer duration of lunchtime compared to recess allowed differences in activity levels between HIGH and LOW groups to emerge [37]. This may have been particularly true as children had more choice as to what types of behaviours to engage in during lunchtime. For example, LOW group children could have spent longer obtaining and eating lunch and socialising with friends compared to HIGH group peers, who may have consumed lunch more quickly to maximise outdoor play time. Two earlier studies of segmented day physical activity reported how pedometer steps accrued during lunchtime contributed 15.4% [6] and 12.6% of total daily steps [5]. Absolute lunchtime activity levels in our study contributed a comparable 13.7% (HIGH) and 12.9% (LOW) to daily MPA, and 15.5% (HIGH) and 13.6% (LOW) to daily VPA suggesting that this segment made a meaningful contribution to daily physical activity levels in both groups.

### After-school

Group differences in MPA (4.5 minutes) and VPA (3.8 minutes) were greatest in the after-school segment. After school provides opportunities for active commuting as well as school-extra-curricular physical activity, which in England often takes the form of structured sports coaching and team practice. Participation in after-school physical activity programmes can make a valuable contribution to children’s physical activity levels [38], fitness status, body composition, and lipid profiles [39]. However, interpreting accelerometer data from the after-school segment is complex due to high day-to-day physical activity variability [7] as children commute home from school using diverse travel modes and at variable times depending on their routines and after-school activity preferences. The hours from 3.30–6.30pm have been described as ‘critical’ for physical activity participation on school days because during this time youth do a variety of activities, including technology-based sedentary activities, physical activity, and homework [40]. A recent study of Australian children also described the post-school period up to 6.30pm as a ‘critical window’ because between these times the greatest differences in MVPA were evident between children classified as high active and low active [30]. The similarities between these findings [30] and our own suggest that the children in the present study engaged in a wide variety of activities after school [6,7]. It is likely that some of the after-school MPA and VPA occurred away from the school environment, and so physical activity may have been partly influenced by parental rules relating to safety [41], parental support for physical activity [42], as well as children’s own activity preferences [40]. After 5pm daylight hours were limited, which most likely restricted outdoor play and active commuting. Thus, the HIGH group’s superior physical activity levels in the after-school segment may have predominantly come through structured physical activity and sports occurring indoors, or on floodlit outdoor facilities.

### Influence of selected child and school-level correlates

BMI z-scores were inversely associated with recess and after-school VPA. This finding is consistent with previous research demonstrating VPA to be independently associated with various measures of adiposity in youth [43]. A one unit shift in BMI z-score may lead to meaningful increases in VPA over the course of a school week, particularly during the after-school period. As VPA is also associated with improved cardiorespiratory fitness [43], strategies to promote higher intensity physical activity, particularly among less active children are warranted from a health perspective. Conversely, out of school MPA was positively associated with BMI z-scores. Other studies have reported equivocal relationships between MPA and adiposity [44] often because of different accelerometer thresholds used to define MPA and VPA [43]. When physical activity intensity has been defined as energy expenditure rather than movement counts, time spent in VPA but not MPA was significantly associated with adiposity [45]. The positive associations between out of school MPA and BMI z-scores may reflect the accelerometer threshold used to represent MPA which is lower than some others in the literature [46,47], and the fact that MPA is accessible and achievable for most children regardless of body size [48].

The positive association between playground area per student and before-school VPA suggests that larger playground areas may have facilitated more vigorous play [11]. However, the beta coefficient for playground area per student was 0.15 m^2^. The interpretation is that for every 1 m^2^ per student increase in playground area, there would be an additional 0.8 minutes of before-school VPA. In absolute terms this converts to an overall increase of 500 m^2^ for a 1 minute increase in before-school VPA. Since the average playground area was 1952 m it is highly unlikely that schools would have the physical space and financial resources to increase playground areas by around 25%. Although the magnitude of the association between playground area per student and lunchtime MPA was larger (β = 0.5) than before-school VPA, the same issues related to practical meaningfulness apply. Thus, whilst the findings highlight significant associations between playground area and physical activity, how meaningful these are in practice is questionable. Similarly, positive associations were observed between student enrolment and MPA before-school and at recess. This might suggest that students attending schools with the greatest enrolments were more likely to engage in MPA before school and at recess, presumably because these schools had more space to accommodate greater student numbers. Once again though, the beta values of 0.002 and 0.004, respectively were too small to be meaningful from a practice or policy perspective.

### Additive effect of segment differences

Over the course of the day the additive effect of the segment differences in activity resulted in the HIGH group accumulating 12.5 minutes more adjusted MVPA than LOW group peers, which equates to 21% of the minimum daily recommended volume of MVPA for health. Some support for the clinical relevance of the 12.5 minutes difference in MVPA is provided by a study which found that a 15 minutes per day difference in MVPA among 12 year olds was associated with up to 12% lower fat mass two years later [49]. Moreover, Andersen et al. observed that children in ascending physical activity quintiles had reduced odds of clustered cardiovascular disease risk [50]. Thus, if the HIGH group at least maintained their level of activity and the LOW group increased theirs, health risks may theoretically be reduced.

This is the first investigation to use accelerometry to examine high and low active children’s physical activity during specific school day segments. Strengths of this study were the specific nature of the segments analysed, high accelerometer compliance rate, and use of multi-level modelling which assessed the effects of a range of child and school level correlates. The models accounted for a substantial amount of segment-specific variance in MPA and VPA, thus including relevant correlates in analyses at the child and school levels can enable the examination of their influence on children’s physical activity levels in different contexts. Study limitations include the sample being from eight schools within one north-west England town where the ethnic diversity of the local population is not representative of other parts of the UK. Therefore, the conclusions may not be generaliseable elsewhere. Furthermore, this was a cross sectional observational study so no conclusions can be made about direction of causality between the predictor and outcome variables. Insufficient data from PE classes did not allow for the inclusion of PE as a discrete segment, and so the study is limited by the lack of PE-specific results. Encouraging class teachers to value PE and not view it a low status subject [51,52] may in future avoid cancellation of PE classes for the sake of convenience. In England the National Curriculum is currently under review with PE recommended as one of the core subjects in the revised curriculum. This positive elevation in PE’s status should increase class teacher accountability relating to PE lesson delivery. IMD scores are an indirect measure of SES at an individual level. Using other measures such as parental education and income may help gain a better understanding of the influence of familial SES on child physical activity. Though accelerometers captured a high proportion of daily physical activity, the inability of these instruments to record upper body movements, water-based activities, and cycling meant that physical activity may have been underestimated in some segments of the day, particularly out of school hours. Moreover, accelerometers provide no information about physical activity behaviours. Combining accelerometry with survey and/or diary approaches such as ecological momentary assessment [40] may provide valuable contextual data to describe physical activity behaviours, and help explain MPA and VPA patterns and differences.

## Conclusions

HIGH active children accumulated more MPA and/or VPA than LOW active children during the before-school, class-time, lunchtime, and after-school segments of the day when analyses were adjusted for a range of correlates. Strategies to increase children’s physical activity could be based around the modifiable correlates identified in this study. Over the course of the day the additive effect of the adjusted segment differences in activity resulted in the HIGH group accumulating 12.5 minutes more MVPA than LOW group peers, which equates to 21% of the minimum daily recommended volume of MVPA. Physical activity promotion strategies targeting low active children during discretionary physical activity segments of the day, and particularly via after-school physical activity programmes may be beneficial.

## Competing interests

The authors declare that they have no competing interests.

## Authors’ contributions

SJF designed the study, supervised the data collection, performed the statistical analysis and drafted and edited the manuscript. AB and HE drafted and edited the manuscript. NDR assisted with the statistical analysis and drafted and edited the manuscript. All authors read and approved the final manuscript.

## Pre-publication history

The pre-publication history for this paper can be accessed here:

http://www.biomedcentral.com/1471-2458/12/406/prepub
